# Microarray analysis of the expression profile of immune-related gene in rapid recurrence early-stage lung adenocarcinoma

**DOI:** 10.1007/s00432-020-03287-7

**Published:** 2020-06-18

**Authors:** Jie Liu, Xiao Yang, Liang Zhang, Bo Yang, Wen Rao, Mengxia Li, Nan Dai, Yuxin Yang, Chengyuan Qian, Lei Zhang, Hualiang Xiao, Dong Wang

**Affiliations:** 1 Cancer Center, Daping Hospital, Army Medical University (Third Military Medical University), No. 10 Changjiang Zhi Rd., Yuzhong Dist., Chongqing, 400042 China; 2 Department of Pathology, Daping Hospital, Army Medical University (Third Military Medical University), No. 10 Changjiang Zhi Rd., Yuzhong Dist., Chongqing, 400042 China

**Keywords:** Microarray, Early-stage lung adenocarcinoma, Rapid recurrence, Differentially expressed genes, Tumor-infiltrating lymphocytes, IL-1β, PTGS2

## Abstract

**Background:**

Although much progress has been made in the diagnosis of early-stage lung adenocarcinoma (ES-LUAD), the prognosis for ES-LUAD patients with rapid recurrence is still poor. Importantly, there is currently no effective and precise method to screen patients who may develop rapid recurrence. Therefore, it is necessary to identify potential differentially expressed genes (DEGs) in ES-LUAD patients with rapid recurrence and non-rapid recurrence.

**Methods:**

Affymetrix GeneChip Human Transcriptome Array was used to identify DEGs between ES-LUAD patients with rapid recurrence and non-rapid recurrence. Rapid recurrence was defined as recurrence-free survival (RFS) ≦ 1 year and non-rapid recurrence was defined as RFS ≧ 3 years. The biological functions of the DEGs were analyzed by GO and KEGG pathway enrichment analyses. The protein–protein interaction (PPI) network of identified DEGs was conducted by STRING and Cytoscape software. The expression level of crucial hub genes and tumor-infiltrating lymphocytes (TILs) was verified by immunohistochemistry (IHC).

**Results:**

A total of 416 DEGs were identified between ES-LUAD patients with and without rapid recurrence. The results of GO analysis revealed that 2 of the top 10 categories in the domain of cellular component, 2 of the top 10 in the domain of molecular function, and 9 of the top 10 in the domain of biological process were functionally related to immunity. The results of KEGG analysis showed that 6 of the top 8 pathways were functionally involved in immune regulation and inflammatory response. The PPI network analysis identified ten crucial nodal protein, including EGFR, MMP9, IL-1β, PTGS2, MMP1, and 5 histone proteins, which constituted 25 key interactions. IL-1β and PTGS2 expression were closely related to immunity and IHC analysis further revealed that low expression of IL-1β and PTGS2 is associated with rapid recurrence. Kaplan–Meier analysis further revealed that LUAD patients with lower IL-1β or PTGS2 expression had a worse RFS. When the TIL density of CD3^+^, CD4^+^, CD8^+^ and CD20^+^ subsets was less than 20%, ES-LUAD patients have a higher probability of rapid recurrence.

**Conclusion:**

There were significant differences in the expression of immune-related genes between patients with rapid recurrence and patient with non-rapid recurrence. Immune-related genes such as IL-1β and PTGS2 and TIL density (20%) play important roles in rapid recurrence of ES-LUAD. This study provided a theoretical basis for distinguishing the two types of patients from an immunological perspective.

**Electronic supplementary material:**

The online version of this article (10.1007/s00432-020-03287-7) contains supplementary material, which is available to authorized users.

## Introduction

Lung cancer is one of the most commonly diagnosed cancer in the world, accounting for 11.6% of the total number of cancer cases, and is also the leading cause of cancer death, accounting for 18.4% of the total number of cancer deaths (Bray et al. 2018). In China, the 5-year survival rate of lung cancer is only 16.1%, and 70% of patients are initially diagnosed with advanced-stage lung cancer with a 5-year survival rate of less than 5% (Zeng et al. 2015). Lung adenocarcinoma (LUAD) is the most common histological type of lung cancer. According to the definition of American Joint Committee on Cancer (AJCC), ES-LUAD refers to tumor size ≦ 5 cm, no lymph node and distant metastasis, also indicated as T1-2N0M0 (IA-IIA).

Currently, the main treatment for ES-LUAD is surgery, and the 5-year overall survival rate is close to 67% (Yanagawa et al. 2013). However, in our clinical practice, some patients with ES-LUAD were relapsed within one year after surgery (16.2%), and the 5-year survival rate was extremely poor, well below the reported 67%, suggesting that ES-LUAD may not be a homogenous but complex disease (unpublished observation). Therefore, it is important to provide more aggressive adjuvant therapy for LUAD patients with rapid recurrence to improve their prognosis. In the past decades, various proteins (Gold et al. 2014), mRNAs (Wistuba et al. 2013), miRNAs (Lu et al. 2012), and DNA methylation (Brock et al. 2008) have been reported to predict postoperative recurrence of early-stage lung cancer, but none of these have been applied for clinical practice. Therefore, it is necessary to understand the molecular mechanism of rapid recurrence of ES-LUAD from a new perspective, and to predict the high-risk ES-LUAD patients who may develop rapid recurrence.

Microarray is a widely used technology to screen differentially expressed genes (DEGs) between cancer and normal tissues and was recently used to study hotspot molecules such as lncRNA (Bach and Lee 2018). However, to the best of our knowledge, there is no study using microarray to investigate the rapid recurrence of ES-LUAD. After hierarchical clustering analysis, we noticed that ES-LUAD patients can be divided into two different categories in the cluster analysis. GO enrichment and KEGG pathway analysis further indicated that these DEGs were mostly functionally related to immunity. Moreover, protein–protein interaction (PPI) network analysis identified many crucial nodal proteins related to immunity function, such as IL-1β, PTGS2, etc. Furthermore, ES-LUAD patients with rapid recurrence had a lower density of CD3^+^, CD4^+^, CD8^+^ and CD20^+^ tumor-infiltrating lymphocytes (TILs) subsets. These results suggested that immune-related genes and TILs participated in the rapid recurrence of ES-LUAD.

## Materials and methods

### Clinical characteristics of patients

The study was conducted in accordance with the guideline of the Helsinki Declaration and was approved by the Ethics Committee of Daping Hospital. All patients in this study underwent surgical treatment at the Daping Hospital from 2011 to 2014, and pathological diagnosis of all patients was confirmed as LUAD. The TNM staging of all patients was T1-2N0M, which was based on the International Union Against Cancer (UICC) 8th Edition TNM lung cancer staging criteria, and was consistent with the definition of ES-LUAD in AJCC. A total of 20 cases with no statistically significant differences in age, gender, smoking history, and degree of differentiation were used for microarray analysis. Among the 20 cases, 8 cases had recurrence within 1 year (classified as rapid recurrence group), and 12 cases had no recurrence within 3 years (non-rapid recurrence group). The clinical characteristics of the 20 patients are shown in Table 1. Microarray was used to examine the expression patterns of 20 patients to identify DEGs between ES-LUAD patients with rapid recurrence and ES-LUAD patients with non-rapid recurrence. On the other hand, another 136 patients were further used to explore the possible molecular of action and involved pathway. Among the 136 patients, 22 cases had recurrence with 1 year, 40 patients had recurrence within 1–3 years, and the remaining 74 patients had no recurrence within 3 years. Patients with recurrence-free survival (RFS) ≦ 1 year were classified as rapid recurrence group, and patients with RFS ≧ 3 years were classified as non-rapid recurrence group. The clinical characteristics of the two groups of patients are shown in Table 2. There were no significant differences in clinical variables between the two groups, including age, gender, smoking history, and degree of differentiation. The clinical data were acquired from medical records and telephone follow-up.

**Table 1:** Clinical characteristics of the patients for gene chip (*n* = 20)

	Rapid recurrence (*n* = 8)	Non-rapid recurrence (*n* = 12)	*P* value
Age	61.88 ± 10.78	57.67 ± 8.74	0.349
Age range			
≥ 60 years	5 (62.5%)	5 (41.7%)	0.65
< 60 years	3 (37.5%)	7 (58.3%)	
Smoking history			
No	4 (50%)	7 (58.3%)	1
Yes	4 (50%)	5 (41.7%)	
Gender			
Male	5 (62.5%)	8 (66.7%)	1
Female	3 (37.5%)	4 (33.3%)	
Differentiation grade			
Low	3 (37.5%)	8 (66.7%)	0.362
Medium or high	5 (62.5%)	4 (33.3%)	

Fisher’s exact test

**Table 2:** Clinical characteristics of the two group of the patients

	Rapid recurrence (*n* = 22)	Non-rapid recurrence (*n* = 74)	*P* value
Age	55.77 ± 9.023	59.23 ± 7.357	0.07
Age range			
≥ 60 years	9 (40.9%)	37 (50%)	0.454
< 60 years	13 (59.1%)	37 (50%)	
Smoking history			
No	13 (59.1%)	48 (64.9%)	0.621
Yes	9 (40.9%)	26 (35.1%)	
Gender			
Male	14 (63.6%)	33 (44.6%)	0.117
Female	8 (36.4%)	41 (55.4%)	
Differentiation grade			
Low	15 (68.2%)	36 (48.6%)	0.107
Medium or high	7 (31.8%)	38 (51.4%)	

Pearson Chi-square test

### Microarray and computational analysis

Microarray analysis was performed using Affymetrix GeneChip^®^ Human Transcriptome Array 2.0 as described in the previous study (Dalma-Weiszhausz et al. 2006). Briefly, total RNAs were extracted and purified separately from 20 paraffin-embedded tumor tissues using QIAGEN's RNeasy^®^ FFPE Kit, reverse-transcribed into cDNA and biotinylated, and then hybridized with GeneChip^®^ Human Transcriptome Array 2.0. The hierarchical clustering analysis was performed by importing the chip data into Transcriptome Analysis Console (TAC) Software 3.0.0. The 416 DEGs were carried out by the Bioconductor package limma using linear modelling and empirical Bayes methods (Ritchie et al. 2015). In this study, DEGs were considered as fold change ≥ 2.0 or ≤ − 2.0 and *P* < 0.05.

### Functional profiling the DEGs by Gene Ontology (GO) and Kyoto Encyclopedia of Genes and Genomes (KEGG)

Gene Ontology (GO) enrichment and Kyoto Encyclopedia of Genes and Genomes (KEGG) pathways analysis were conducted to clarify the functional roles of DEGs. Enriched functional categories of gene–ontology associations were performed by clusterProfiler R software. The KEGG analysis was performed using the Database for Annotation, Visualization and Integrated Discovery (DAVID) database (https://www.david.ncifcrf.gov/home.jsp) (Sherman et al. 2007). Adjusted *P* values less than 0.05 was considered statistically significant.

### Analysis of the protein–protein interaction (PPI) network

All 416 DEGs were imported to The Search Tool for the Retrieval of Interacting Genes/Proteins (STRING) database (https://www.string-db.org/) for PPI network analysis (Szklarczyk et al. 2019). A composite score > 0.4 was considered statistically significant. The node proteins obtained from the STRING database were then imported into Cytoscape software to obtain a PPI network map. The cytoHubba plug-in of Cytoscape software was used to analyze and identify the top 10 crucial node proteins and the top 10 crucial genes in the PPI network.

### Immunohistochemistry (IHC)

The paraffin-embedded tissues were cut into approximately 3 μm sections and spread on glass slides. The sections were deparaffinized, rehydrated, and then antigen retrieval by high pressure heating streamer using Tris/EDTA buffer (heat-induced epitope retrieval). After leaving to cool, the sections were treated with 3% H_2_O_2_—methanol solution for 10 min to eliminate endogenous peroxidase activity. The sections were then washed with PBS and incubated overnight at 4 °C with a primary antibody: IL-1β (sc-32294, 1:200, Santa Cruz Biotechnology), PTGS2 (ZA-0515, ZSGB-BIO), CD3 (ZM-0417, ZSGB-BIO), CD4 (ZA-0519, ZSGB-BIO), CD8 (ZA-0508, ZSGB-BIO) or CD20 (ZA-0293, ZSGB-BIO). After washing with PBS, the sections were incubated with a horseradish peroxidase-conjugated secondary antibody (Rabbit HRP EnVision TM+, Dako, Denmark) for 30 min at 37 °C. After washing with PBS, the sections were incubated with 3,3-diaminobenzidine (DAB) substrates for 3 min, counterstained with hematoxylin for 2 min, and finally dehydrated. The brown color indicated positive staining. For the negative control, the steps were the same except that the primary antibody was omitted.

For calculating the density of tumor-infiltrating lymphocytes (TILs) subsets (CD3^+^, CD4^+^, CD8^+^ and CD20^+^ TILs), five microscope fields were randomly selected, and the number of positive stained cells and nucleated cells in each field was calculated by Image-Pro Plus software 6.0. IHC staining score of IL-1β and PTGS2 was determined based on the staining intensity of cancer cells and graded as follows: negative, score 0; weak, score 1; moderate, score 2; and strong, score 3. Scoring was independently performed by two experienced pathologists.

### Statistics

All statistical analyses were performed using GraphPad Prism version 8.0.2 (GraphPad, La Jolla, CA). Comparisons of proportions were used Fisher’s exact test or Pearson Chi-square test, as appropriate. In the Kaplan Meyer analysis, comparisons of survival curves were used Log-rank (Mantel–Cox) test. And *P* < 0.05 was considered statistically significant.

## Results

### Overview of DEGs

**Fig. 1:**
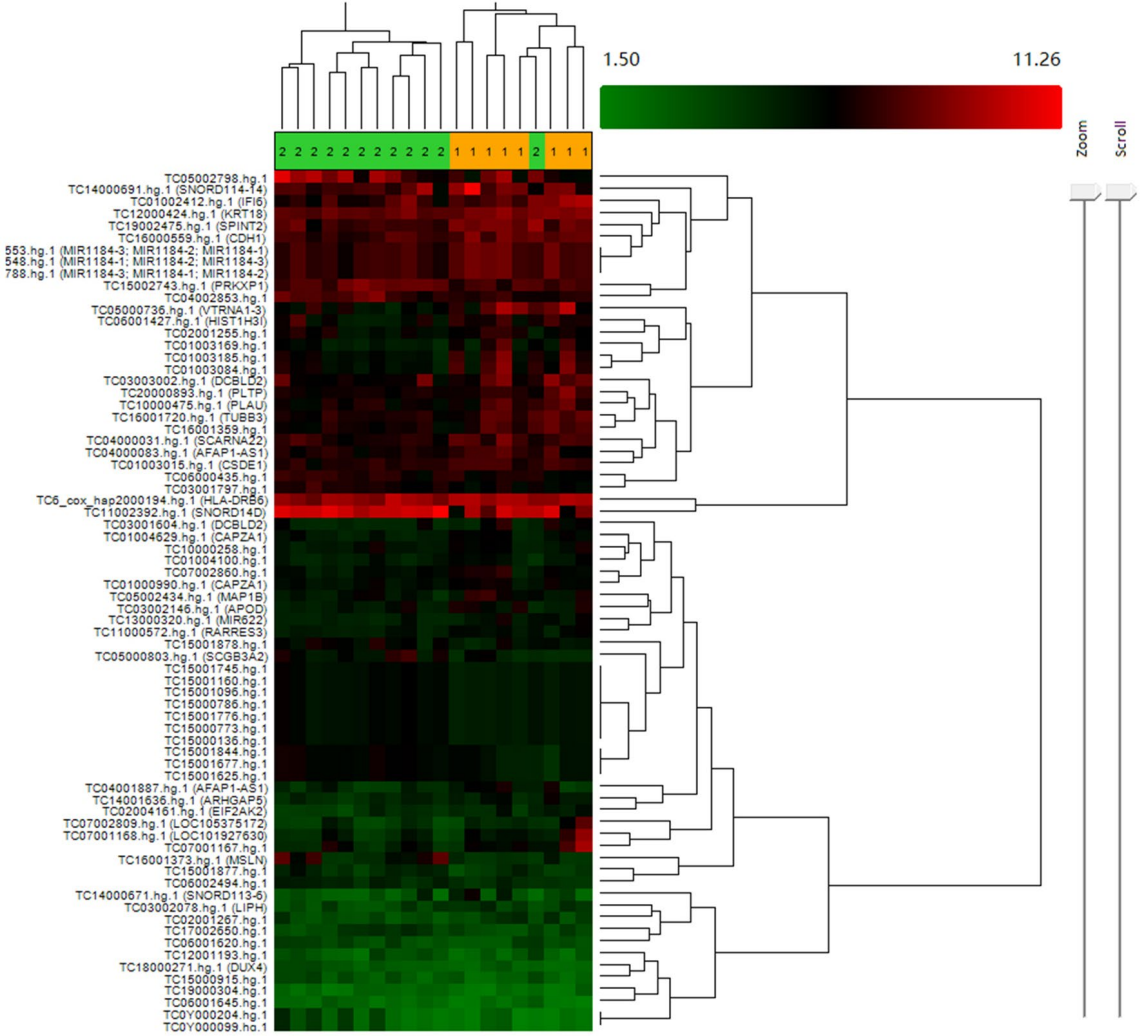
Hierarchical clustering analysis of DEGs in ES-LUAD patients with/without rapid recurrence. The number 1 in the orange background indicated ES-LUAD patients with rapid recurrence. There were 8 patients in this group. The number 2 in the green background indicated 12 ES-LUAD patients without rapid recurrence, but only 11 patients were classified in this group. The remaining 1 case was classified as group 1. Red, high expression; green, low expression

Figure 1 shows a clustering analysis of 72 significant DEGs (Table S1) microarray data from ES-LUAD patients with rapid recurrence or non-rapid recurrence. The results indicated that the two groups of patients can basically be divided into two different categories. Using the Bioconductor package limma, a total of 416 DEGs (Table S2) were obtained between rapid recurrence group and non-rapid recurrence group. Of these DEGs, 156 and 260 genes were up-regulated and down-regulated in the rapid recurrence group, respectively (fold change ≧ 2.0 or ≦ − 2.0; *P* < 0.05). The top 10 DEGs with the highest fold change are listed in Table 3.

**Table 3:** Top 10 differentially expressed genes in rapid recurrence compared with Non-rapid recurrence

Up			Down	
Gene	Foldchange		Gene	Foldchange
XAGE1E	13.96		XIST	30.4
MT1A	7.64		CEACAM5	21.27
HLA-DQA1	7.3		S100A9	11.77
MMP13	7.28		IGJ	10.31
DSP	7.26		GDF15	9.42
MT1L	6.71		SPINK1	9.13
SNORD14D	6.39		PTGS2	9.02
MMP1	6.19		PLAT	8.96
COL12A1	5.26		IGLV7-43	7.93
AREG	4.09		IGLV7-46	7.89

### Gene Ontology (GO) term enrichment analysis of 416 DEGs

**Fig. 2:**
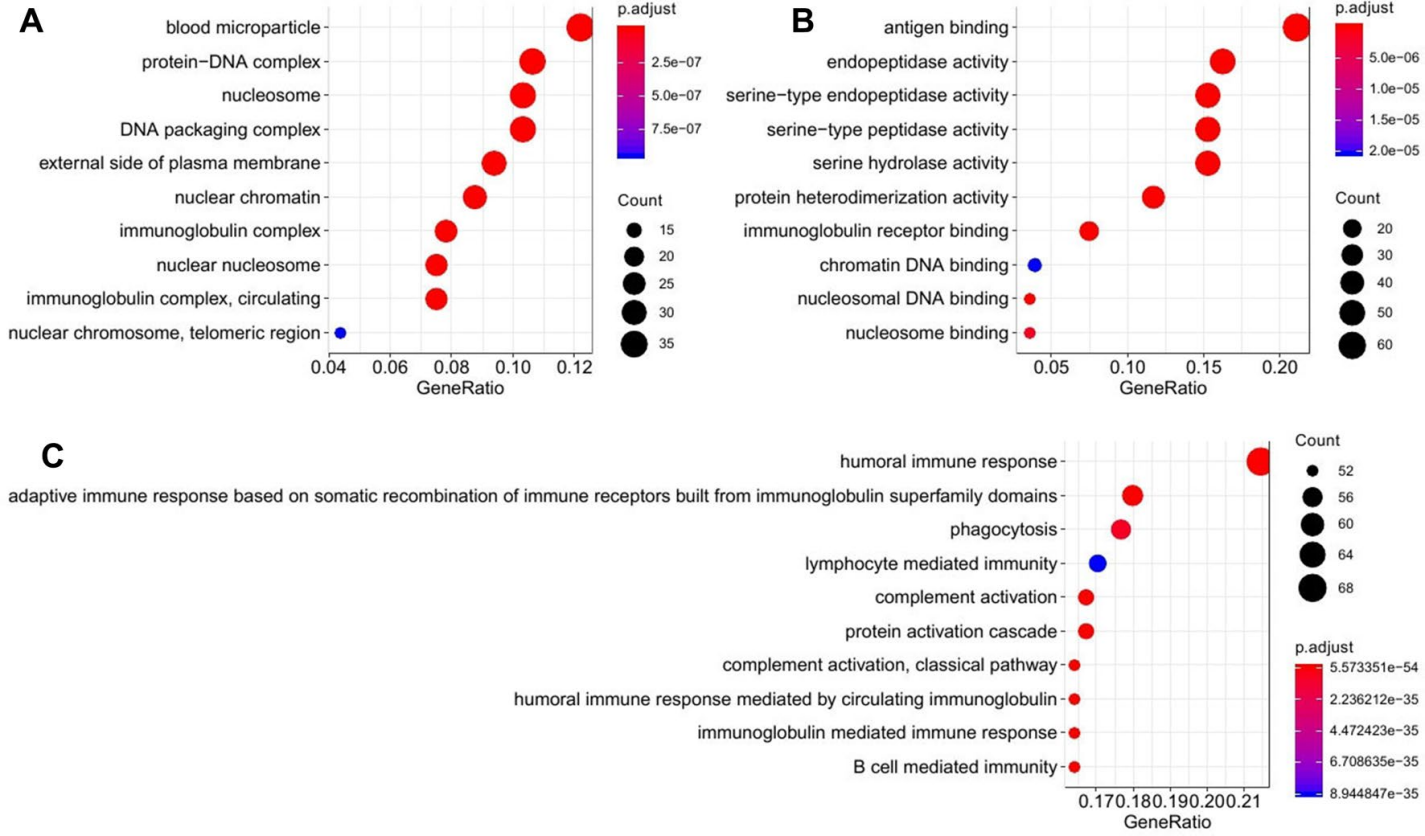
GO enrichment analysis of DEGs between the two groups. **a** The top 10 GO categories in the domain of cellular components by analyzing 416 DEGs. Two of these categories were related to immunity. **b** The top 10 GO categories in the domain of molecular function. Two of these categories were related to immunity response. **c** The top 10 GO categories in the domain of biological process. Nine of these categories were related to immunity or inflammation. GeneRatio: number of DEGs annotated to the GO category/total number of DEGs

The Gene Ontology (GO) covers three domains, including cellular component, molecular function, and biological process. The 416 DEGs were classified into differential functional categories according to the GO term enrichment analysis. And the top 10 functional categories from 416 DEGs in each domain are shown in Fig. 2. In the domain of cellular component, two of the top 10 categories were related to immunity, including immunoglobulin complex (GO:0019814) and immunoglobulin complex, circulating (GO:0042571). In the domain of molecular function, two of the top 10 categories were related to immune response, including antigen binding (GO:0003823) and immunoglobulin receptor binding (GO:0034987). Remarkably, in the domain of biological process, nine of the top 10 categories were related to the immune process, including humoral immune response (GO:0006952), adaptive immune response based on somatic recombination of immune receptors built from immunoglobulin superfamily domains (GO:0002460), phagocytosis (GO:0006909), lymphocyte mediated immunity (GO:0002449), complement activation (GO:0006956), complement activation, classical pathway (GO:0006958), humoral immune response mediated by circulating immunoglobulin (GO:0002455), and immunoglobulin mediated immune response (GO:0016064), and B cell mediated immunity (GO:0019724).

### Kyoto Encyclopedia of Genes and Genomes (KEGG) pathway analysis

**Fig. 3:**
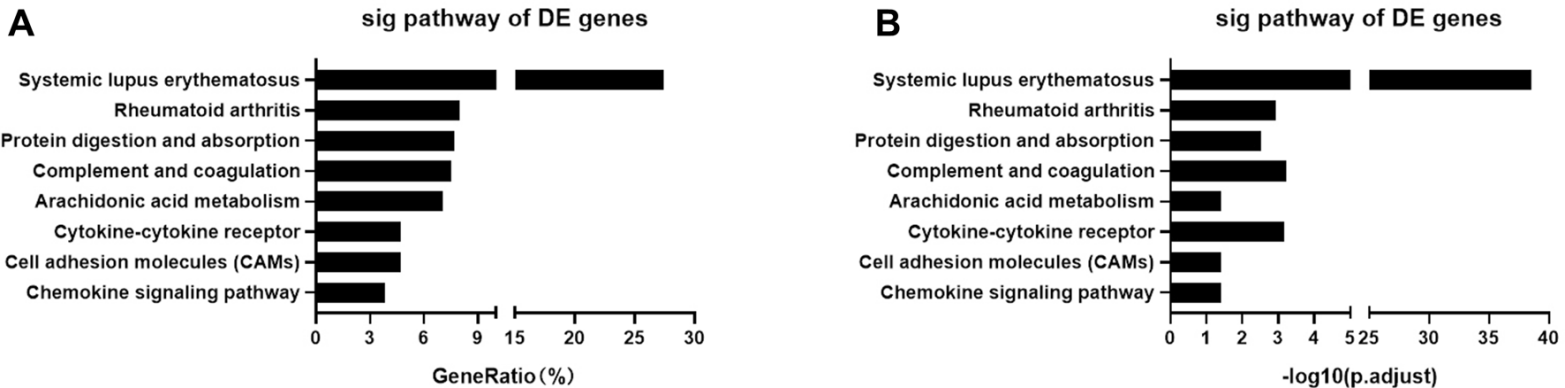
KEGG pathway analysis of DEGs between the two groups. The top 8 pathways significantly enriched in DEGs were listed. Of these, 6 of the 8 pathways were functionally related to immunity or inflammation. The *x*-axis represents the GeneRatio (**a**) and the -log10 of the enrichment *P* value (**b**). The *y*-axis represents the KEGG pathway. GeneRatio: number of DEGs annotated to the KEGG pathway/total number of DEGs

To understand the possible pathways of the 416 upregulated or downregulated DEGs in the cell, these genes were mapped to the KEGG pathway. As shown in Fig. 3, the DEGs were highly clustered in eight metabolic or signaling pathways. It is worth noting that six of the top 8 pathways were functionally involved immune regulation or inflammatory disease, including systemic lupus erythematosus, rheumatoid arthritis, complement and coagulation, arachidonic acid metabolism, cytokine-cytokine receptor, chemokine signaling pathway.

### PPI network

**Fig. 4:**
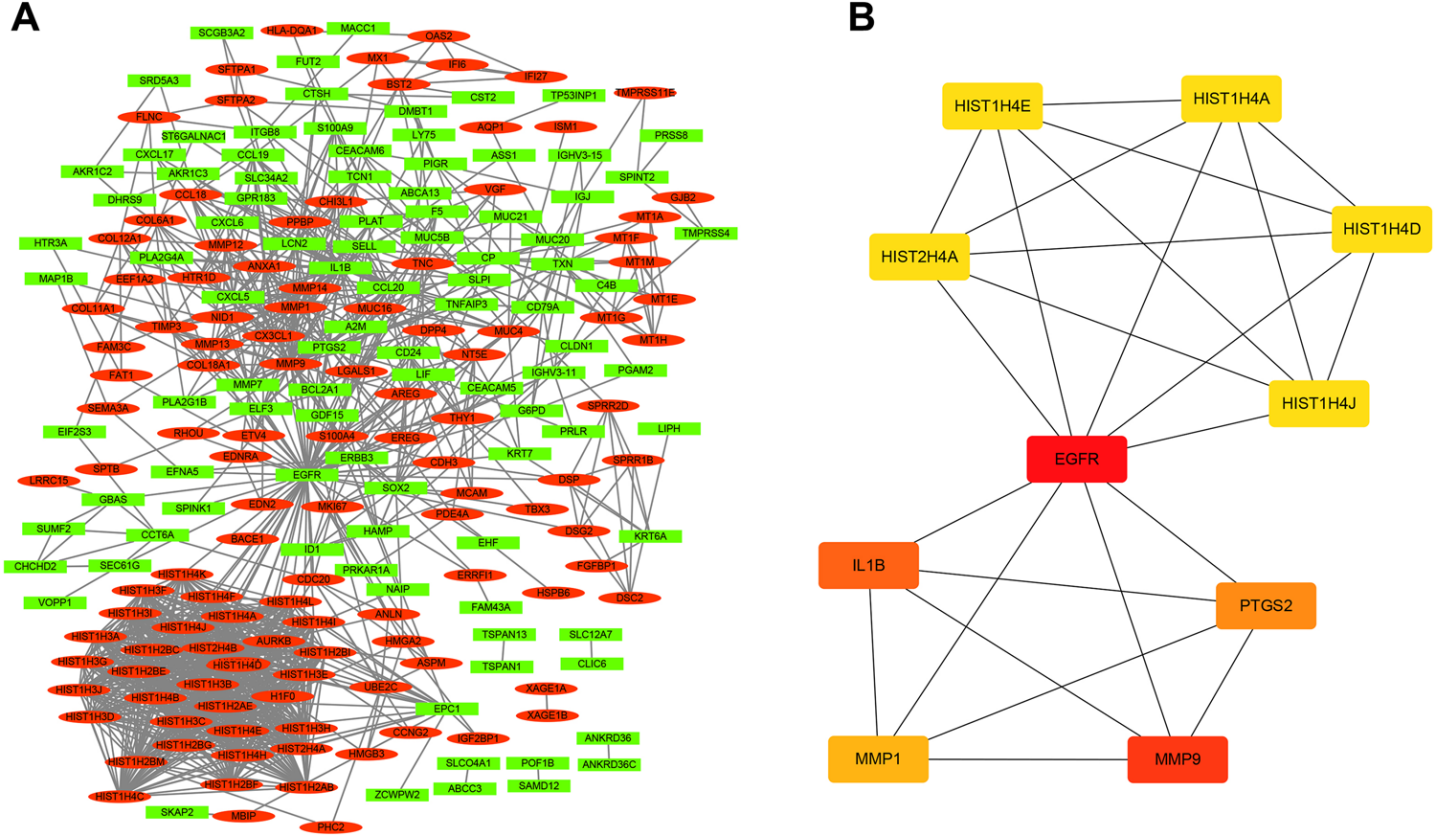
PPI network analysis for DEGs for rapid recurrence LUAD samples. **a** The PPI network consisted of 216 nodes (nodal proteins) and 1149 edges (interactions between proteins). In addition, the green rectangle indicated down-regulated protein and the red oval indicated up-regulated protein. **b** PPI network showed the top 10 hub genes, including EGFR, MMP9, PTGS2, IL1B, MMP1, HIST1H4E, HIST2H4A, HIST1H4A, HIST1H4D, and HIST1H4J. The 10 key genes constituted 25 interactions with each other. The darker the color, the more critical the effect

For the 416 identified DEGs identified from rapid recurrence samples, 216 nodal proteins and 1149 interacted edges were obtained for PPI network analysis based on the STRING database. As shown in Fig. 4a, 117 proteins were up-regulated (red oval) and 99 proteins were down-regulated (green rectangle). Topological analysis was performed using cytoHubba, a Java plug-in for Cytoscape software, to analyze 216 nodal proteins and identify 10 key nodal proteins, including epidermal growth factor receptor (EGFR), matrix metalloproteinase 9 (MMP9), interleukin 1β (IL-1β), cyclooxygenase2 (PTGS2), matrix metalloproteinase 1 (MMP1), and 5 histones (HIST1H4E, HIST2H4A, HIST1H4A, HIST1H4D and HIST1H4J). These nodal proteins were considered to be key proteins in the whole network and ultimately constituted a total of 25 key interactions (Fig. 4b).

### Immunohistochemistry (IHC) analysis of the two immune-related genes IL-1β and PTGS2

**Fig. 5:**
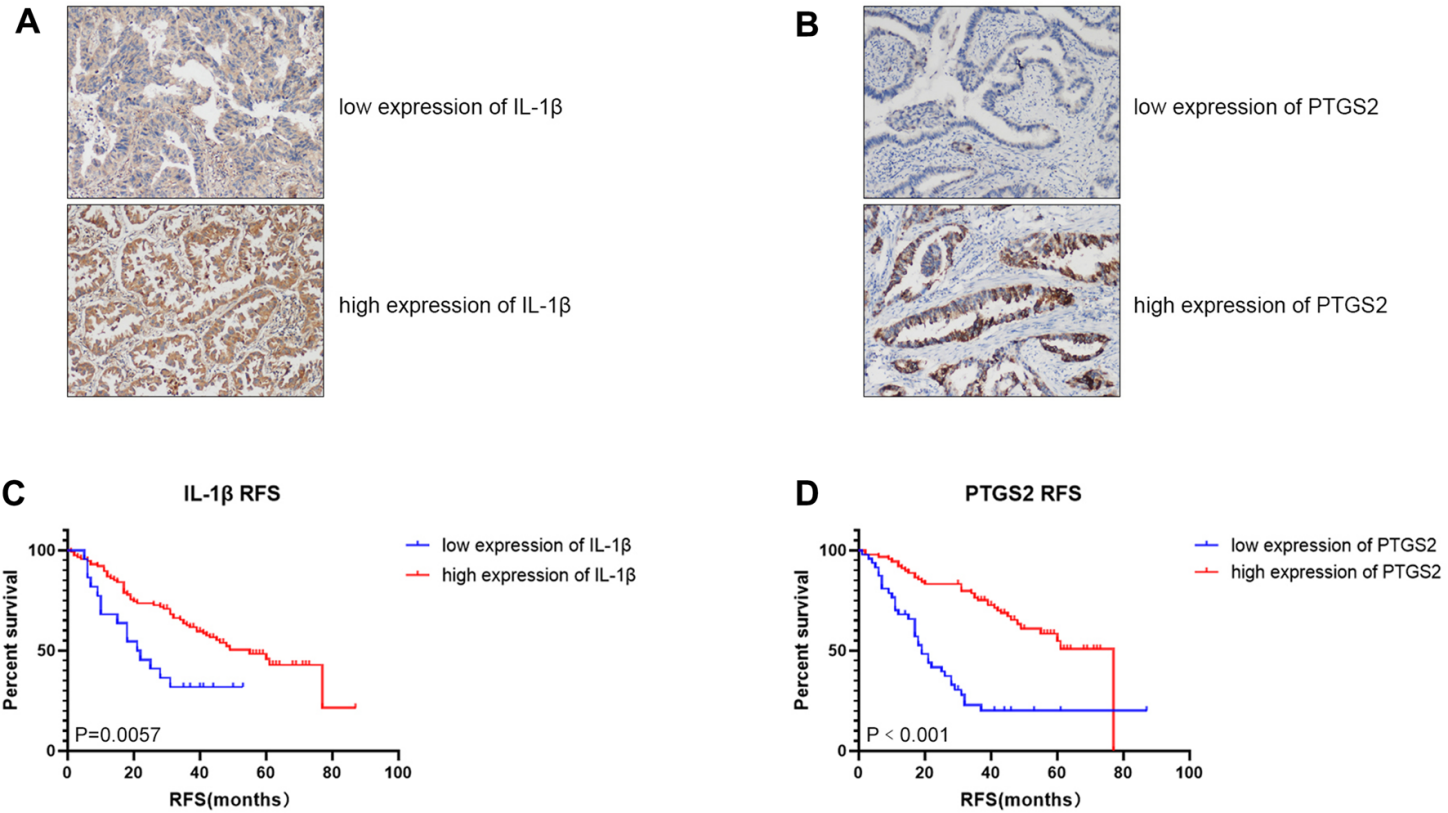
Recurrence-free survival in ES-LUAD patients. Representative images showed the expression level of IL-1β (**a**) or PTGS2 (**b**) in clinical samples from ES-LUAD patients (× 100). The Kaplan–Meier analyses of RFS for ES-LUAD patients with low or high expression of IL-1β (**c**) or PTGS2 (**d**). Patients with higher IL-1β or PTGS2 had a better RFS

The expression of IL-1β (Fig. 5a) and PTGS2 (Fig. 5b) in 136 patients with early-stage LUAD were examined by IHC analysis. In consistent to the microarray results, the expression of IL-1β and PTGS2 were relatively low in the rapid recurrence ES-LUAD tissues (Table 4). Compared to the non-rapid recurrence group, the proportion of low IL-1β expression in the rapid recurrence group was significantly higher (31.8% vs 8.1%, *P* = 0.012). The proportion of low PTGS2 in the rapid recurrence group was also significantly higher than that in the non-rapid recurrence group (68.2% vs 10.8%, *P* < 0.001). Furthermore, survival analyses were performed to explore the individual prognostic significance of IL-1β and PTGS2 in ES-LUAD patients using IHC staining (Fig. 5a, b) and Kaplan–Meier analysis (Fig. 5c, d). The results showed a significant difference in recurrence-free survival (RFS) between high and low expression of IL-1β (Fig. 5c) and PTGS2 (Fig. 5d). The median RFS was 21.5 months and 55.0 months in low and high expression of IL-1β, respectively (*P* = 0.0057). As to the PTGS2, the median RFS was 19.0 months and 77.0 months in low and high expression of PTGS2 (*P* < 0.001), respectively.

**Table 4:** the protein expression of IL-1β and cox-2 between the two groups

	Expression	Rapid recurrence (*n* = 22)	Non-rapid recurrence (*n* = 74)	*P*
IL-1β	Low	7 (31.8%)	6 (8.1%)	0.012
	High	15 (68.2%)	68 (91.9%)	
cox-2	Low	15 (68.2%)	8 (10.8%)	0.000
	High	7 (31.8%)	66 (89.2%)	

Pearson Chi-square test

### The relationship between tumor-infiltrating lymphocytes subsets and rapid recurrence in ES-LUAD patients

**Fig. 6:**
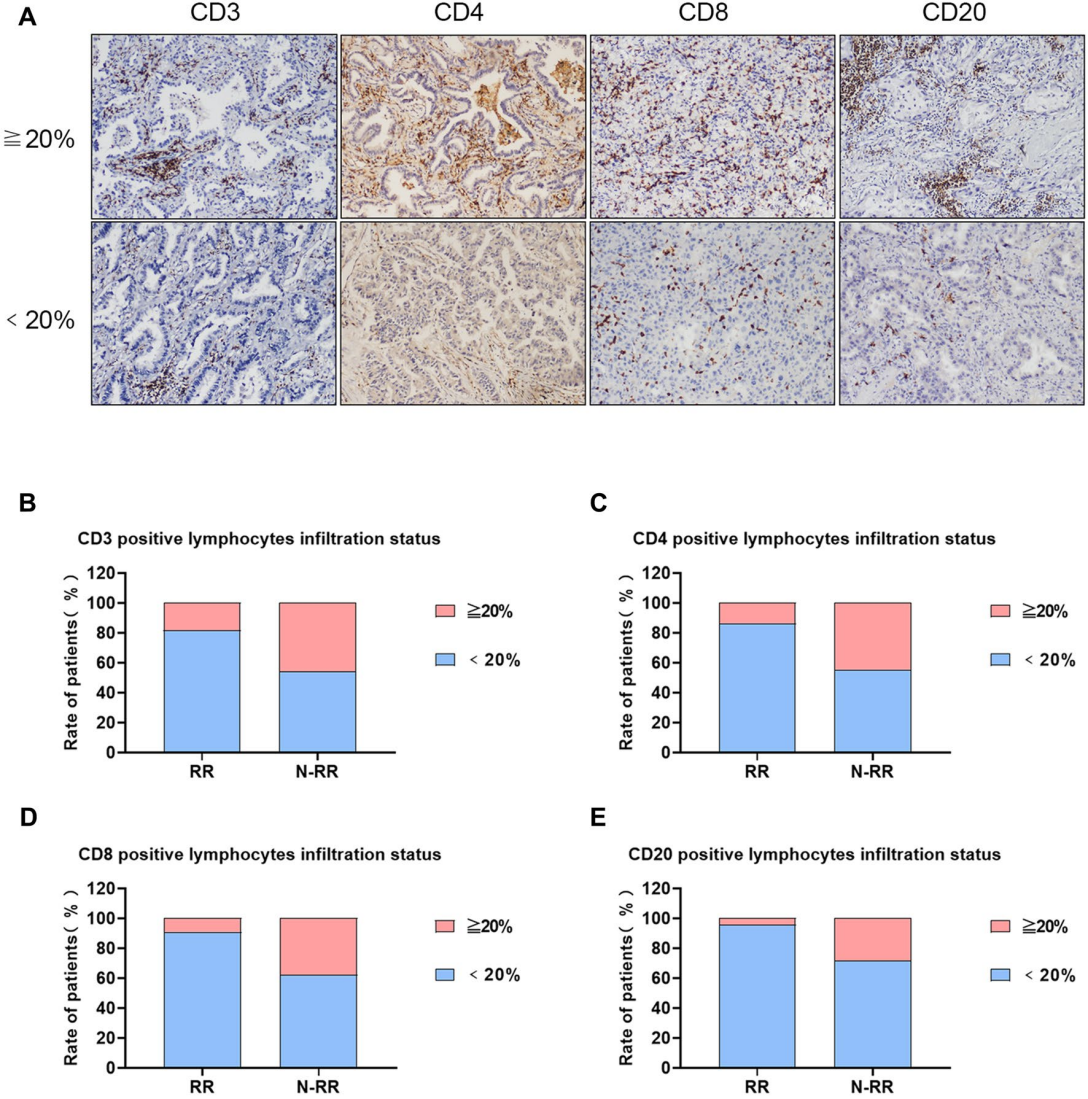
Tumor-infiltrating T and B lymphocytes in the rapid and non-rapid recurrence patients. **a** Representative images of CD3^+^, CD4^+^, CD8^+^ and CD20^+^ TILs by IHC analysis (× 100). The upper panels showed the TILs density (tumor lymphocyte infiltration rate) ≧ 20%, and the below panels showed TILs density < 20%. The densities of CD3^+^ (**b**), CD4^+^ (**c**), CD8^+^ (**d**), and CD20^+^ (**e**) TILs subsets in rapid recurrence and non-rapid recurrence groups were analyzed and displayed. When the TILs density ≧ 20%, the proportion of patients in the rapid recurrence group was significantly lower than that in the non-recurrence group: CD3^+^(18.2% vs. 45.9%), CD4^+^ (13.6% vs. 44.6%), CD8^+^ (9.1% vs. 37.8%) and CD20^+^ (4.5% vs. 28.4%)

To further investigate the association between the density of tumor-infiltrating lymphocytes (TILs) subsets and rapid recurrence, tumor lymphocyte infiltration rates were determined and analyzed by IHC staining the infiltrating lymphocyte subsets. The TILs density was classified as high (≧ 20%) and low (< 20%). And the IHC images of TILs density of CD3^+^, CD4^+^, CD8^+^ and CD20^+^ subsets are shown in Fig. 6a. Herein, the CD3^+^, CD4^+^, CD8^+^, and CD20^+^ subsets represented the total T cells, CD4^+^ helper T cells, CD8^+^ cytotoxic T cells, and B cells, respectively. As shown in Fig. 6b–e and Table 5 (*P* < 0.05, Chi-square test), the density of all 4 TILs subsets were significantly different between rapid recurrence (*n* = 22) and non-rapid recurrence groups (*n* = 74). The results indicated that when the TILs density was less than 20%, ES-LUAD patients have a higher probability of rapid recurrence.

**Table 5:** Tumor-infiltrating lymphocytes between the two groups

TILs	Infiltration ratio (%)	Rapid recurrence (*n* = 22)	Non-rapid recurrence (*n* = 74)	*P* value
CD3	< 20	18 (81.8%)	40 (54.1%)	0.019
	≧ 20	4 (18.2%)	34 (45.9%)	
CD4	< 20	19 (86.4%)	41 (55.4%)	0.008
	≧ 20	3 (13.6%)	33 (44.6%)	
CD8	< 20	20 (90.9%)	46 (62.2%)	0.011
	≧ 20	2 (9.1%)	28 (37.8%)	
CD20	< 20	21 (95.5%)	53 (71.6%)	0.020
	≧ 20	1 (4.5%)	21 (28.4%)	

Pearson Chi-square test

## Discussion

The histological subtypes of LUAD are closely related to the prognosis. Warth et al. reported that among the lepidic, acinar, papillary, micropapillary and solid predominant subtypes, the lepidic predominant subtype had the longest overall survival (OS) (Warth et al. 2012). Ujiie et al. reported that the solid predominant subtype is an independent predictor of recurrence in patients with stage I LUAD (Ujiie et al. 2015). In addition to histological subtypes, some biomarkers were also used to predict the postoperative recurrence of early-stage lung cancer (Gold et al. 2014; Wistuba et al. 2013; Lu et al. 2012; Brock et al. 2008), but they do not really distinguish patients with rapid recurrence. In our clinical practice, ES-LUAD patients had extremely poor prognosis after rapid recurrence. Therefore, it is necessary to identify these patients who will have rapid recurrence and provide them with more aggressive treatment. Thus, in this study, 20 ES-LUAD patients were classified into rapid recurrence group (RFS ≦ 1 year) and non-rapid recurrence group (RFS ≧ 3 years), and DEGs between the two groups were analyzed by microarray. 72 DEGs were clustered by the Transcriptome Analysis Console (TAC) Software 3.0.0. We found that 20 patients could be basically divided into two categories: all 8 patients with rapid recurrence were classified into one category, and 11 of 12 patients with non-rapid recurrence were classified into another category. This supported the hypothesis that from a genetic perspective, ES-LUAD patients with rapid recurrence or non-rapid recurrence may be two different disease subtypes. Thus, screening ES-LUAD patients for rapid recurrence is theoretically feasible. Next, we used the Bioconductor package limma to identify DEGs because it has been a popular choice for gene discovery through differential expression analyses of microarray over the past decade (Ritchie et al. 2015). A total of 416 DEGs were obtained by this method. It should be pointed out that the DEGs identified through the Transcriptome Analysis Console (TAC) Software 3.0.0. and the Bioconductor package limma are not completely consistent, which may be caused by different algorithms and statistical methods.

To further understand the functions of these DEGs involved in cells, GO enrichment and KEGG pathway analysis were performed on these 416 DEGs. GO and KEGG analyses are commonly used to demonstrate function enrichment of the DEGs between tumor and non-tumor tissues (Yuan et al. 2018), and can also be used to predict oncogenes (Xing et al. 2016). Thus, a functional enrichment analysis was first conducted to analyze 416 DEGs identified from rapid recurrence group and non-rapid recurrence group. In GO analysis, whether it is the cellular component aspect, the molecular function aspect or the biological process aspect, the functions of these DEGs were obviously related to immunity. In detail, in the top 10 categories of the three GO aspects, 2 of top 10 in cell components category, 2 of top 10 in the molecular function category, and 9 of 10 in the biological process category were all functionally related to immunity. Interestingly, all the three GO aspects contained humoral immunity-related terms (GO:0019814, GO:0034987, GO:0006952, etc.), suggesting that humoral immunity played a special role in the rapid recurrence of ES-LUAD. In the KEGG analysis, these DEGs were also found to be functionally involved in immune-related or inflammation-related pathways, that is, there were 6 of the top 8 pathways. Of interest, systemic lupus erythematosus (SLE) ranked first. SLE is due to the inability of individual’s immune system to distinguish between self and non-self-antigens, resulting in the production of antibodies against self-antigens and triggering an over-active inflammatory response (Hui-Yuen et al. 2016). Since impaired B cells are typical characteristics of SLE and B cells are the main cells involved in humoral immunity (Bakshi et al. 2018), this implies that in ES-LUAD patients, the occurrence of rapid recurrence may be closely related to humoral immunity. In summary, the GO enrichment results indicated that there is a large difference in the expression of immune-related genes between ES-LUAD patients with rapid recurrence and non-rapid recurrence.

We also found that among 416 DEGs, 156 genes were up-regulated and 260 genes were down-regulated. Xu et al. suggested that key genes play important biological functions, rather than those with the highest expression difference (Xu et al. 2018). Thus, the PPI network analysis was conducted using the STRING database to identify the key genes and finally obtained 216 key nodal proteins. Subsequently, 10 key genes (EGFR, MMP9, IL1B, PTGS2, MMP1, HIST1H4E, HIST2H4A, HIST1H4A, HIST1H4D, and HIST1H4J) were obtained by Cytoscape software. Interestingly, similar to the results of GO functional enrichment analysis, we found that many of these 10 key genes were functionally related to immunity or inflammation, such as IL1B and PTGS2.

IL-1β is a potent proinflammatory cytokine and was originally discovered as the main endogenous pyrogen that induces the synthesis of prostaglandin. It was later confirmed that IL-1β has multiple functions, including T cell activation and cytokine production, B cell activation and antibody production, promoting Th17 differentiation of T cells, etc. (Schett et al. 2016). Studies have also shown that sustained induction of IL-1β enhanced the intensity of the inflammatory response and generated an inflammatory microenvironment to promote the initiation and development of tumors (Bhat et al. 2014; Dinarello 2006). Furthermore, high levels of IL-1β in tumors and serum were associated with higher tumor grades and increased invasion of breast, pancreatic cancer, and myelogenous leukemia, and were also associated with poor prognosis (Setrerrahmane and Xu 2017). PTGS2, also known as COX-2, encodes the rate-limiting enzyme cyclooxygenase, which converts arachidonic acid to prostaglandins. Unlike the constitutive expression of COX-1, PTGS2 is an inducible enzyme that is activated in response to extracellular stimuli, such as growth factors and proinflammatory cytokines. Some investigators demonstrated that PTGS2 is overexpressed in a variety of epithelial malignancies, such as lung, breast, pancreas, colon, and esophagus, and is usually associated with poor prognosis (Hida et al. 1998; Hwang et al. 1998; Okami et al. 1999; Ogino et al. 2008; Takatori et al. 2008). Although high expression of either IL-1β or PTGS2 is believed to be associated with poor prognosis in many types of tumors, our microarray data showed different results indicating that IL-1β and PTGS2 expression were significantly lower in patients with rapid recurrence than in patients with non-rapid recurrence. In other words, IL-1β and PTGS2 may act as protective factors to reduce the occurrence of rapid recurrence. Because our results of IL-1β and PTGS2 were inconsistent with some literature reports, we further performed IHC analysis to examine the expression of IL-1β and PTGS2 in another group of 136 ES-LUAD patients. Consistent with our microarray data, IHC results also indicated that the expression of IL-1β and PTGS2 in the rapid recurrence group were significantly lower than in the non-rapid recurrence group. Our survival analyses of 136 patients further supported the results that patients with high expression of IL-1β or PTGS2 have a better RFS.

IL-1 promotes the expansion of natural killer (NK) cells and CD4^+^ CD8^+^ T cells by combining with IL2 (Ben Aribia et al. 1950). IL-1β down-regulated TGF-β-induced Foxp3 expression, thereby inhibiting the differentiation of regulatory T cells (Ikeda et al. 1950). These reports illustrate the potential role of IL-1β in antitumor immunity. In Allen’s in vivo studies, IL-1β also showed protective effects in mouse models of chemical colitis and colon cancer (Allen et al. 2010). Therefore, it can be reasonably assuming that IL-1β acts as a protective factor for preventing rapid recurrence in ES-LUAD patients. From the perspective of inflammation and tumors, our reasonable theory is that early inflammation can exert anti-tumor effects, but long-term persistent chronic inflammation induces tumorigenesis. Supporting this theory, high levels of IL-1 in chronic inflammation was found to promote tumor development by driving sustained NF-κB activation and MAPK activity (Bent et al. 2018). In another aspect, the LUAD patients in this study were in early tumor stage rather than late/advanced tumor stage. Thus, IL-1β may not promote tumor development through sustained long-term inflammatory stimulation, but may inhibit tumors through an anti-tumor immunity. As to PTGS2, PTGS2 encoded prostaglandin-endoperoxide synthase 2 is responsible for the production of prostaglandins, which plays a key role in the inflammatory response (Ricciotti and FitzGerald 2011). As mentioned above, inflammation is a double-edged sword that promotes or inhibits tumor development. Therefore, similar to IL-1β, PTGS2 will not promote tumor progression through sustained inflammatory stimuli in early-stage LUAD. Moreover, studies have shown that high level of PTGS2 can inhibit tumor growth and migration by regulating 8‐HOA, another derivative of PTGS2 (Hashemi Goradel et al. 2019). In summary, 2 immune-related genes of the 10 key genes, IL-1β and PTGS2, were identified in this study. They were functionally closely related to immunity or inflammatory response, suggesting a significant immune difference between ES-LUAD patients with rapid recurrence and non-rapid recurrence.

It is worth noting that EGFR is the central linker to IL1B and PTGS2, but it has not been studied in this paper because our research focuses on immune-related genes. Overexpression of EGFR in NSCLC tumors has been reported in many series, and the reported results of EGFR (over)expression range from 43 to 89% (Hirsch et al. 2003). The prognostic significance of EGFR overexpression in NSCLC has also been studied. Some studies showed that EGFR overexpression was associated with shortened survival, while others found no prognostic implication of EGFR overexpression (Hirsch et al. 2003). In our microarray data, we found that EGFR was lower in the rapid recurrence group (foldchange = − 2.11) on the level of mRNA, but the relationship between EGFR protein level and recurrence needs further research.

In addition to IL-1β and PTGS2, TILs may also be closely related to rapid recurrence in ES-LUAD patients. NSCLCs are frequently associated with prominent TILs and other inflammatory cells (Rekhtman et al. 2013). Several studies suggested that there was a positive correlation between patient survival, treatment response, and the number of TILs (Schalper et al. 2015; Donnem et al. 2015; Brambilla et al. 2016). In stage 1A NSCLC patients, the levels of intratumoral TILs are positively associated with improved RFS (Horne et al. 2011). In addition, the spatial distribution of TILs in tumors was related to the recurrence of early-stage NSCLC (Corredor et al. 2019). However, there is no report on whether TILs are involved in the rapid recurrence of ES-LUAD patients. Therefore, IHC was performed to determine the density of TILs subsets in the rapid recurrence group and the non-rapid recurrence group, and the results showed that the number of infiltrating total T cells (CD3^+^), CD4^+^ helper T cells (CD4^+^), CD8^+^ cytotoxic T cells (CD8^+^), and B cells (CD20^+^) were all significantly reduced in the rapid recurrence group. Since IL-1β can promote the proliferation and differentiation of activated B cells (Lipsky et al. 1950) and stimulate T cell replication (North et al. 1988), it is likely that IL-1β play a crucial role in the regulation the number of TILs in the recurrence of ES-LUAD patients. In this study, IL-1β expression was significantly lower in the rapid recurrence group than in the non-rapid recurrence group. It is possible that in non-rapid recurrence of ES-LUAD patients, higher level of IL-1β may promote the proliferation or replication of TILs within tumors to exert anti-tumor immunity. This warrants further investigation.

The results of the functional enrichment analysis and the identified key DEGs and TILs clearly revealed that there are significant differences in the immune level between two groups of ES-LUAD patients. Therefore, in ES-LUAD patients, rapid recurrence and non-rapid recurrence may be two different disease subtypes and have their unique phenotypes in immunity. Furthermore, our research may help broaden the application of lung cancer immunotherapy. In the past, immune checkpoint inhibitors have achieved amazing results in non-small cell lung cancer (NSCLC), whether in mono (Peters et al. 2018) or combination immunotherapy (Borghaei et al. 2019), but only for advanced NSCLC. Even recently, the combination of Neoadjuvant and consolidation immuno-oncology therapy has been propose as an effective treatment for stage III NSCLC (Yeh et al. 2018). Currently, an important predictive biomarker for lung cancer immunotherapy is PD-L1. It is reported that patients with PD-L1 overexpressing have a 67–100% response rate; whereas for PD-L1 negative, the response rate is about 0–15% (Patel and Kurzrock 2015). However, for early-stage LUAD (IA-IIA), the current treatment is still surgery-based, and immunotherapy is not recommended. Because the prognosis of patients with rapid recurrence is extremely poor, and their immune status are unique, these DEGs are very likely to become the first potential targets or biomarkers other than PD-L1 for immunotherapy in early-stage lung cancer in the future.

In conclusion, our findings provided a possible mechanism for the rapid recurrence of ES-LUAD patients and a theoretical basis for distinguishing ES-LUAD patients, who may develop rapid recurrence from an immunological perspective. Moreover, these DEGs will be the most likely potential targeting gene in future immunotherapy for ES-LUAD patients with rapid recurrence.

## Electronic supplementary material

Below is the link to the electronic supplementary material.

Supplementary file1 (XLSX 11 kb)

Supplementary file2 (XLSX 41 kb)
